# Post-COVID Gastrointestinal Symptoms and Disorders of Gut-Brain Interaction: A Focus on Persistent Diarrhea

**DOI:** 10.7759/cureus.117111

**Published:** 2026-09-28

**Authors:** Fauz Saheb

**Affiliations:** 1 Molecular and Cellular Biology, Johns Hopkins Bloomberg School of Public Health, Baltimore, USA

**Keywords:** covid-19, diarrhea, disorders of gut-brain interaction, functional bowel disorders, gut microbiome, irritable bowel syndrome, long covid, post-covid syndrome, post-infectious ibs

## Abstract

Persistent gastrointestinal (GI) symptoms after SARS-CoV-2 infection include diarrhea, abdominal pain, dyspepsia, constipation, nausea, and other disorders of gut-brain interaction (DGBIs), but these entities should not be treated as interchangeable. In a meta-analysis of long-COVID studies, any GI symptom was reported in approximately 22% of patients and diarrhea in 10%, although heterogeneity was very high. In a large US Veterans Affairs cohort, post-acute COVID-19 was associated with incident diarrhea (hazard ratio {HR}: 1.58, 95% CI: 1.52-1.65; excess burden 7.99 per 1,000 persons at one year) and irritable bowel syndrome (HR: 1.54, 95% CI: 1.28-1.86). Prospective Rome IV cohorts report lower absolute rates of formally diagnosed DGBIs; one study found persistent DGBI symptoms in 6.6% at six months, with functional diarrhea in 2.2% and irritable bowel syndrome (IBS) in 2.5% at three months. Mechanistic evidence supports intestinal involvement during acute infection and demonstrates associations with epithelial/angiotensin-converting enzyme 2 (ACE2) perturbation, immune activation, barrier dysfunction, altered microbial taxa, and impaired microbial metabolite production. However, persistent viral RNA or antigen, dysbiosis, and gut-lung signaling remain proposed or associative mechanisms rather than established causes of chronic diarrhea. Treatment evidence specific to post-COVID functional diarrhea is sparse. A large synbiotic trial improved a broad “gastrointestinal upset” domain in post-acute COVID syndrome, but did not enroll a defined chronic diarrhea or Rome-positive population; probiotic and fecal microbiota transplantation trials showing GI effects were largely conducted during acute COVID-19. Therefore, current care should first exclude organic and medication-related causes, then apply phenotype-based management extrapolated from established diarrhea-predominant irritable bowel syndrome (IBS-D) and functional-diarrhea practice while clearly acknowledging the limited post-COVID-specific evidence. Standardized definitions, pre-infection GI data, appropriate controls, functional microbiome measures, and dedicated randomized trials are priorities for future research.

## Introduction and background

Post-acute gastrointestinal manifestations of SARS-CoV-2 infection span a broad spectrum. Patients may report persistent or new diarrhea, abdominal pain, nausea, altered appetite, dyspepsia, reflux, constipation, or other bowel symptoms after the acute infection [1-4]. These symptom reports are clinically important, but they are not synonymous with a functional bowel disorder or disorders of gut-brain interaction (DGBI). A DGBI is a positive clinical diagnosis based on a characteristic symptom pattern and accepted criteria after reasonable consideration of alternative organic disease; a symptom such as post-COVID diarrhea is initially a descriptive phenotype, not a diagnosis [5,6].

Acute GI involvement provides biologic plausibility for later bowel dysfunction. Diarrhea, nausea/vomiting, abdominal pain, and anorexia were documented in early hospitalized and ambulatory cohorts, and viral RNA was detectable in stool in a substantial subset of patients [7-12]. Acute findings, however, should not be automatically extrapolated to chronic post-COVID symptoms because the relevant populations, time points, treatments, and competing causes differ.

This review focuses on persistent or new-onset diarrhea after COVID-19 while placing it within the wider post-COVID GI spectrum. Diarrhea is emphasized because it is common enough to generate a measurable post-acute burden, overlaps clinically with functional diarrhea and diarrhea-predominant irritable bowel syndrome (IBS-D), and has direct implications for hydration, diagnostic evaluation, work capacity, and quality of life [1,3,4]. The review distinguishes broad post-COVID GI symptoms from Rome-defined DGBIs, separates acute from post-acute mechanistic and therapeutic evidence, and identifies where current management is based on post-COVID trials versus extrapolation from established gastroenterology practice.

## Review

Methods of the narrative review

This manuscript is a narrative review rather than a systematic review or meta-analysis. For the peer-review revision, a targeted literature update was performed on September 19, 2026, using PubMed/MEDLINE for English-language publications from January 1, 2020, through September 19, 2026. The following two complementary search concepts were used: (1) (COVID-19 OR SARS-CoV-2) AND (long COVID OR post-COVID OR post-acute COVID-19 OR post-acute sequelae) AND (gastrointestinal OR diarrhea OR diarrhoea OR irritable bowel syndrome OR functional diarrhea OR disorders of gut-brain interaction OR microbiome OR dysbiosis); and (2) (COVID-19 OR SARS-CoV-2) AND (gastrointestinal OR diarrhea OR diarrhoea) AND (angiotensin-converting enzyme 2 {ACE2} OR permeability OR cytokine OR microbiome OR metabolite OR probiotic OR synbiotic OR fecal microbiota transplantation). We hand-checked reference lists of key systematic reviews, major cohort studies, randomized trials, and clinical guidelines for additional relevant publications. Eligible evidence included adult human observational studies, randomized controlled trials, systematic reviews/meta-analyses, and major clinical guidance relevant to epidemiology, mechanisms, diagnosis, or treatment. Pediatric-only studies, animal-only studies, non-peer-reviewed preprints, isolated case reports, and studies without a relevant GI or post-infectious outcome were not prioritized. Acute-COVID studies were retained only when they directly informed GI mechanisms or intervention safety/efficacy and were explicitly identified as acute evidence. Duplicate publications were reconciled manually. Screening and synthesis were conducted at the narrative-review level; no independent dual-reviewer screening, formal risk-of-bias instrument, PRISMA flow diagram, or original quantitative pooling was performed. Effect estimates from the strongest studies are reported where available, and the limitations of this approach are addressed below.

Terminology and scope

For clarity, this review uses “post-COVID GI symptoms” as an umbrella description for GI complaints occurring after acute infection. “Post-COVID diarrhea” refers descriptively to persistent, recurrent, or new loose/watery stools after COVID-19 and does not imply a functional etiology. “Functional diarrhea” and “IBS-D” are Rome-defined DGBIs; IBS-D requires recurrent abdominal pain as a central feature, whereas functional diarrhea is characterized by loose/watery stools without predominant abdominal pain. “Post-infectious IBS” (PI-IBS) describes IBS that begins after an acute infectious illness in a person without pre-existing IBS. COVID-19 may act as an infectious trigger for PI-IBS, but a distinct “post-COVID IBS” mechanism has not been established [5,6,13,14]. Table 1 summarizes these operational distinctions among diarrhea-related post-COVID phenotypes.

**Table 1:** Operational distinction among diarrhea-related post-COVID phenotypes. DGBIs: disorders of gut-brain interaction; IBS: irritable bowel syndrome; PI-IBS: post-infectious IBS; IBS-D: diarrhea-predominant irritable bowel syndrome

Term	Practical definition	Pain central?	Time course	Relationship to COVID-19
Post-COVID diarrhea	Descriptive symptom phenotype: persistent, recurrent, or new loose/watery stools after COVID-19.	No	May be transient or chronic; does not by itself satisfy Rome criteria.	Can reflect infection, medication effects, bile-acid diarrhea, inflammatory disease, malabsorption, DGBI, or other causes.
Functional diarrhea	Rome-defined DGBI with recurrent loose/watery stools without predominant abdominal pain or bothersome bloating.	Predominant pain absent	Chronic symptom pattern required under Rome criteria.	COVID-19 can temporally precede symptoms, but the diagnosis remains based on phenotype and exclusion of important alternatives.
IBS-D	IBS phenotype with recurrent abdominal pain associated with defecation and/or change in stool frequency/form, with diarrhea-predominant stool pattern.	Yes	Rome chronicity criteria apply.	May occur after COVID-19, but pain-based IBS criteria must be met.
PI-IBS	IBS developing after a documented acute infectious illness in a person without prior IBS.	Yes	Persistent post-infectious syndrome after the acute illness resolves.	COVID-19 can be a potential infectious trigger; it is not yet clear that the resulting phenotype is biologically distinct from PI-IBS after other infections.
Other DGBI/post-COVID GI symptoms	Functional dyspepsia, bloating, constipation, nausea/vomiting syndromes, reflux-like symptoms, etc.	Varies	Varies by diagnosis.	Should not be labeled a functional bowel disorder solely because symptoms follow COVID-19.

Epidemiology of post-COVID GI symptoms and DGBIs

Quantitative Estimates

The magnitude of post-COVID GI burden depends strongly on outcome definition. In a systematic review and meta-analysis of long COVID, the pooled frequency of any GI symptom was 22% (95% CI: 10%-41%); diarrhea was 10% (95% CI: 4%-23%), dyspepsia 20% (95% CI: 6%-50%), and IBS 17% (95% CI: 6%-37%). The very high heterogeneity (I² approximately 96%-98% for several outcomes) limits use of these pooled percentages as patient-level predictions [3].

Large administrative cohorts quantify relative risk but may not establish a Rome-defined functional disorder. In the US Veterans Affairs study of 154,068 COVID-19 survivors, compared with contemporary controls, diarrhea had an HR of 1.58 (95% CI: 1.52-1.65) and an estimated excess one-year burden of 7.99 per 1,000 persons; IBS had an HR of 1.54 (95% CI: 1.28-1.86), and functional dyspepsia an HR of 1.36 (95% CI: 1.22-1.51) [1]. In a Bronx health-system cohort followed up to 3.5 years, 6.34% of 35,102 COVID-19 patients developed a new GI diagnosis versus 5.70% of controls; the adjusted HR for the composite GI outcome was 1.18 (95% CI: 1.12-1.25) [2]. These datasets demonstrate excess post-acute GI healthcare diagnoses but include outcomes broader than functional bowel disease.

Prospective studies applying Rome IV criteria generally report lower absolute rates. Golla et al. followed 320 COVID-19 cases, 320 household controls, and 280 seronegative controls: 11.3% of cases reported new functional gastrointestinal disorder (FGID)/DGBI symptoms at one month, 8.4% at three months, and 6.6% at six months; at three months, IBS occurred in 2.5% and functional diarrhea in 2.2%, while no healthy controls developed FGID through six months [4]. In a separate case-control study, 5.3% of 280 COVID-19 patients met IBS criteria at six months, compared with one of 264 controls [15]. A 2024 meta-analysis estimated pooled post-COVID IBS incidence at 12%, but the pooled RR versus non-COVID controls was 1.23 (95% CI: 0.50-3.01), illustrating uncertainty generated by heterogeneous definitions, follow-up, and study design [16]. A 2025 multicenter analysis identified post-COVID DGBIs in 27 of 599 eligible patients (4.5%), again emphasizing that formally defined DGBIs represent a narrower group than all post-COVID GI symptoms [17]. Table 2 summarizes representative epidemiological studies and their quantitative findings.

**Table 2:** Representative epidemiological studies of post-COVID GI outcomes. VA: Veterans Affairs; DGBIs: disorders of gut-brain interaction; PI-DGBI: post-infection disorders of gut-brain interaction; IBS: irritable bowel syndrome

Studies	Design/population	Follow-up	Key quantitative result	Interpretation/limitation
Xu et al. (2023) [1]	US VA retrospective cohort; 154,068 COVID survivors plus >5.6 million contemporary controls	Post-acute through 1 year	Diarrhea HR: 1.58 (1.52-1.65), excess burden 7.99/1,000; IBS HR: 1.54 (1.28-1.86)	Large controlled cohort; predominantly VA population; coded outcomes do not equal Rome-confirmed DGBI
Choudhury et al. (2022) [3]	Systematic review/meta-analysis; 50 studies	Variable long-COVID definitions	Any GI symptom 22% (10%-41%); diarrhea 10% (4%-23%); IBS 17% (6%-37%)	Very high heterogeneity and mixed symptom definitions
Golla et al. (2023) [4]	Prospective cohort; 320 COVID, 320 household controls, 280 seronegative controls	1, 3, 6 months; Rome IV	DGBI symptoms 11.3%, 8.4%, 6.6%; at 3 months IBS 2.5%, functional diarrhea 2.2%	Prospective Rome-based ascertainment; relatively modest sample
Ghoshal et al. (2022) [11]	Prospective case-control; 280 COVID, 264 historical controls	6 months	IBS 5.3%, uninvestigated dyspepsia 2.1%, overlap 1.8%; one control developed IBS	Supports PI-DGBI signal; historical control design and pandemic-era factors may confound
Changela et al. (2024) [2]	Retrospective Bronx health-system cohort; 35,102 COVID, 682,594 controls	Up to 3.5 years	New composite GI diagnosis 6.34% versus 5.70%; adjusted HR: 1.18 (1.12-1.25)	Long follow-up and adjustment; composite includes organic and functional diagnoses
Wang et al. (2024) [18]	Systematic review/meta-analysis; 10 studies	6-12 months and variable	Pooled IBS incidence 12%; pooled RR versus controls 1.23 (0.50-3.01)	Wide CI and heterogeneity; does not prove excess IBS risk
Marasco et al. (2025) [19]	Post-hoc analysis of prospective multicenter cohort; 599 eligible	Baseline, 1, 6, 12 months	Post-COVID DGBI in 27 (4.5%)	Updated multicenter evidence; small number of post-COVID DGBI cases

Diarrhea Versus Other GI Outcomes and Symptom Duration

Diarrhea is a reproducible acute and post-acute symptom, but it is not uniformly the dominant late GI complaint. Acute meta-analyses primarily involving hospitalized patients estimated diarrhea around 7-8%, whereas long-COVID studies include dyspepsia, appetite disturbance, abdominal pain, constipation, and reflux-like symptoms at comparable or higher frequencies [3,8-11]. These estimates should not be combined across disease phases. A symptom present for several weeks after an infection may still represent resolving post-infectious illness; a Rome-defined DGBI requires a chronic, pattern-based diagnosis. Prospective studies showing decreasing symptom prevalence from one to six months support this distinction [4,15].

Demographic, Geographic, and Clinical Heterogeneity

The most consistent prospective risk signal is the presence of GI symptoms during acute infection, with some cohorts also identifying greater acute disease severity and psychological comorbidity as predictors of later DGBI [4,15]. Evidence for a universal effect of sex, age, vaccination status, or variant is less consistent. Large database studies can adjust for measured comorbidities but incompletely capture diet, antibiotic exposure, access to care, health-seeking behavior, and pre-infection microbiome composition [1,2]. Variant and vaccination eras also overlap with changes in hospitalization practices and treatment, making causal attribution difficult. Accordingly, demographic or geographic differences should be interpreted as context-dependent rather than as a single reproducible risk profile.

Pathophysiology and Mechanistic Evidence

Figure 1 summarizes a plausible mechanistic framework while emphasizing that no single causal chain has been established. Many data supporting ACE2 perturbation, cytokine activation, permeability changes, and dysbiosis derive from acute infection or broad post-acute syndromes rather than patients with chronic diarrhea meeting Rome criteria [7,18-31].

**Figure 1:**
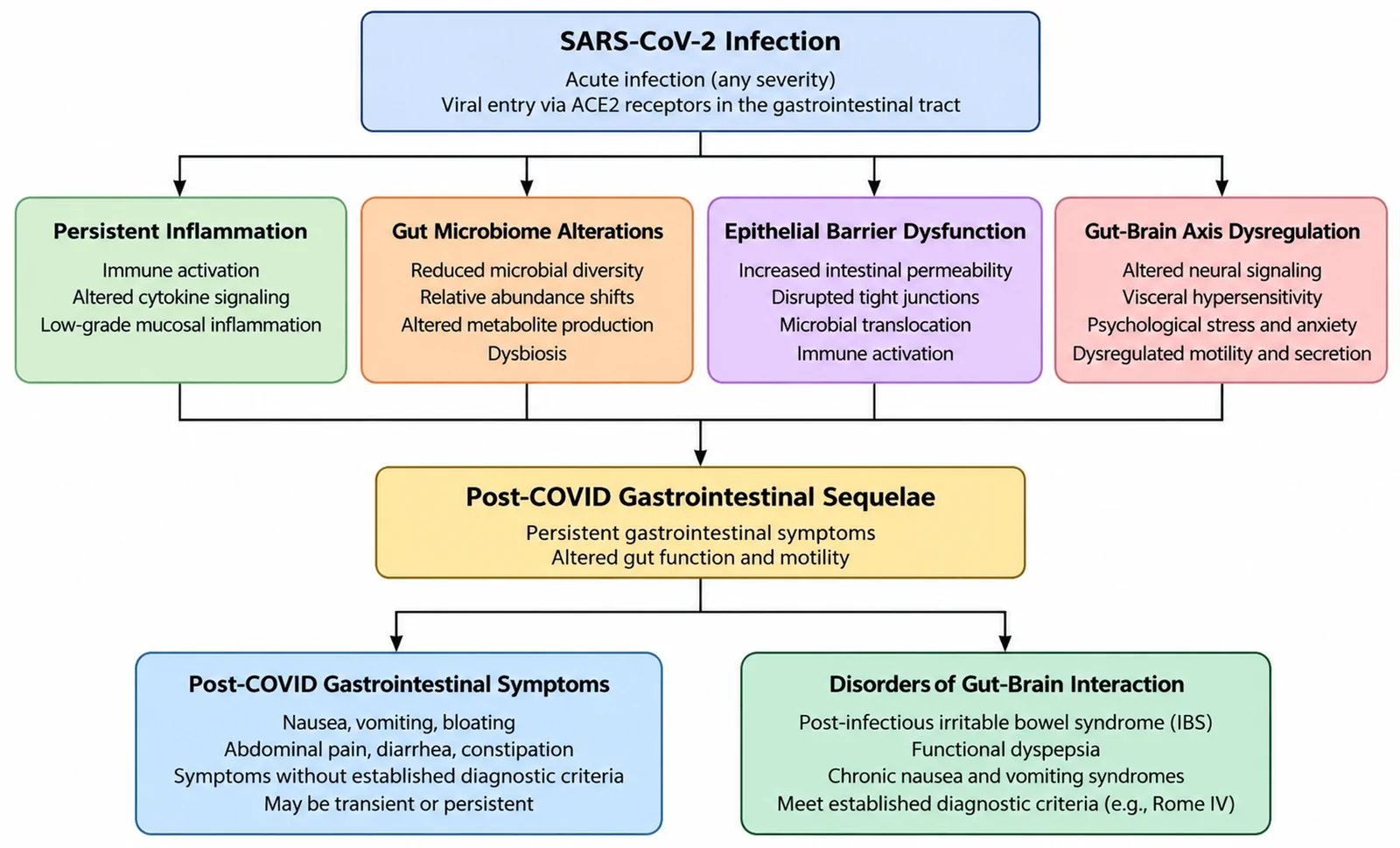
Proposed mechanisms linking SARS-CoV-2 infection with persistent diarrhea and DGBI phenotypes. The figure separates biologically plausible associations from proven causality; persistent RNA/antigen and gut-lung pathways are explicitly qualified. ACE2: angiotensin-converting enzyme 2; DGBI: disorders of gut-brain interaction

Intestinal Infection, ACE2, and Viral Persistence

The intestinal epithelium expresses ACE2 and entry-associated proteases, and experimental and clinical data support intestinal involvement during acute SARS-CoV-2 infection [7,20,23,27,29]. Altered ACE2-related signaling may affect amino-acid transport, antimicrobial peptide expression, epithelial integrity, and local immune regulation. These observations provide biologic plausibility but do not establish that ACE2 disruption persists as the cause of chronic diarrhea.

Viral persistence requires careful terminology. In a longitudinal study of 113 people with mild-to-moderate COVID-19, fecal SARS-CoV-2 RNA was detected in 12.7% at four months and 3.8% at seven months, demonstrating prolonged RNA shedding but not necessarily replication-competent virus [21]. In an endoscopic study of 46 patients with inflammatory bowel disease approximately seven months after COVID-19, gut mucosal viral RNA was detected in 32 and nucleocapsid protein in 24; importantly, investigators were unable to culture virus from tissue [22]. This selected IBD cohort is not equivalent to a population with post-COVID functional diarrhea. Thus, RNA detection, antigen persistence, viable virus, and causal persistence-related diarrhea are distinct claims; only the first two have substantial human evidence in selected cohorts.

Immune Activation and Mucosal Barrier Dysfunction

Acute COVID-19 can produce cytokine and chemokine activation, epithelial injury, and markers compatible with increased intestinal permeability [7,18,26,27]. Barrier disruption can expose mucosal immune cells to microbial products and alter secretion or motility. However, direct evidence linking persistent cytokine activation or permeability abnormalities specifically to chronic post-COVID functional diarrhea is limited. Available post-acute studies often evaluate broad multisystem symptoms, small selected cohorts, or surrogate biomarkers rather than a well-phenotyped diarrhea endpoint. The most defensible interpretation is therefore that immune and barrier abnormalities are plausible contributors whose causal magnitude in chronic diarrhea remains uncertain.

Microbiome, Microbial Taxa, and Metabolites

Acute COVID-19 microbiome studies repeatedly report depletion of potentially beneficial commensals. In a two-hospital cohort, *Faecalibacterium prausnitzii*, *Eubacterium rectale*, and bifidobacteria were underrepresented and remained reduced in serial samples collected up to 30 days after disease resolution [30]. In a prospective PACS cohort, patients with symptoms at six months had higher *Ruminococcus gnavus *and* Bacteroides vulgatus *andlower* F. prausnitzii*; butyrate-producing *Bifidobacterium pseudocatenulatum* and *F. prausnitzii* showed inverse correlations with PACS [28]. These PACS symptoms were predominantly non-GI, so the findings cannot be assumed to explain chronic diarrhea.

Functional microbial changes may be as important as taxonomic shifts. In 66 antibiotic-naive COVID-19 patients, pathways for short-chain fatty acid (SCFA) and L-isoleucine biosynthesis were impaired and remained abnormal beyond 30 days after recovery; measured fecal SCFAs were also lower, with associations to severity and inflammatory markers [18]. GI-specific post-acute data are more limited but emerging; a study that followed 983 antibiotic-naive patients with mild COVID-19 identified distinct oral/gut microbial and serum metabolomic patterns in a small subset with GI symptoms at three months [31]. Bile-acid and tryptophan alterations have also been reported in acute/severe COVID-19 studies, but direct evidence connecting a specific bile-acid signature to persistent post-COVID functional diarrhea remains insufficient [25].

Confounding is a major limitation. Antibiotics, hospitalization, diet, geography, medications, acute disease severity, and baseline health can independently alter microbiome composition. Antibiotic-naive designs reduce one source of confounding, but most studies do not fully control diet, pre-infection microbiota, socioeconomic context, or all medication exposures [18,31]. Chronic diarrhea itself can alter microbial composition, creating reverse causation. Microbiome findings should therefore be interpreted as associations until longitudinal, pre-infection, functionally characterized studies demonstrate directionality.

Gut-Brain and Proposed Gut-Lung Interactions

The gut-brain axis integrates enteric neural, central nervous system, immune, endocrine, and microbial signaling. Post-infectious inflammation, stress, sleep disruption, altered diet, and changes in activity can interact with visceral sensitivity and motility without implying that symptoms are purely psychological [5,6,32]. This framework is well aligned with DGBI biology. By contrast, gut-lung communication is biologically plausible and relevant to systemic inflammation, but its specific role in chronic post-COVID diarrhea has not been established; it should be considered a proposed or incompletely defined pathway rather than a proven mechanism [25,26]. Figure 1 summarizes the proposed mechanisms linking SARS-CoV-2 infection with persistent gastrointestinal symptoms and disorders of gut-brain interaction. Table 3 summarizes the proposed mechanisms and the strength and limitations of current human evidence.

**Table 3:** Proposed mechanisms and strength of current evidence. ACE2: angiotensin-converting enzyme 2; IBD: inflammatory bowel disease; PACS: post-acute COVID-19 syndrome; SCFA: short-chain fatty acid; DGBI: disorders of gut-brain interaction; PI-IBS: post-infection IBS

Mechanism	Human evidence	Disease phase	Relevance	Key caveat
Enterocyte/ACE2 effects	Human intestinal involvement and biologic plausibility; acute clinical and experimental evidence [7,20,23,27,29]	Mostly acute	Can affect epithelial transport, barrier, and local immunity	Persistence of ACE2-related dysfunction as a cause of chronic diarrhea is not established
Fecal viral RNA/mucosal antigen	RNA shedding months after infection [21]; mucosal RNA/protein in selected IBD cohort [22]	Post-acute, selected cohorts	Supports tissue exposure/persistence hypothesis	RNA/antigen ≠ replication-competent virus; tissue culture negative in the IBD study; causal link to chronic diarrhea unproven
Immune/barrier dysfunction	Cytokines, inflammatory markers, permeability/barrier signals [7,26,27,29]	Mainly acute/broad post-acute	Plausible effects on secretion, motility, sensory signaling	Few data from well-phenotyped chronic post-COVID diarrhea cohorts
Microbiome taxa	PACS associations with *R. gnavus, B. vulgatus, F. prausnitzii* [28]; reduced *F. prausnitzii/E. rectale*/bifidobacteria in acute/early recovery [30]	Acute+broad PACS	Potential links to barrier and immune tone	Diet, antibiotics, geography, severity, and reverse causation are major confounders
SCFAs/metabolites	Reduced microbial SCFA biosynthesis and fecal SCFAs after recovery [18]; GI-long-COVID microbiome/metabolome associations [31]	Post-acute; limited GI-specific data	Functional readouts may be more informative than taxonomy	Association does not prove symptom causality; replication needed
Gut-brain signaling	Strong biologic framework from DGBI/PI-IBS literature [5,6,14,32]	Established in DGBI; COVID-specific magnitude uncertain	Visceral sensitivity, motility, stress/immune integration	Not specific to SARS-CoV-2
Gut-lung signaling	Mechanistic rationale and systemic inflammatory links [25,26]	Proposed	Possible cross-organ immune signaling	Specific contribution to chronic diarrhea is poorly established

Clinical Phenotypes and Relationship to PI-IBS

Post-COVID bowel dysfunction may represent several different clinical pathways as follows: slow resolution of acute infectious diarrhea, an organic or medication-related disorder unmasked during/after infection, a new DGBI triggered by infection, or exacerbation of a pre-existing bowel disorder. The overlap with PI-IBS is substantial because both can follow infection and involve altered barrier function, low-grade immune activation, dysbiosis, visceral hypersensitivity, and gut-brain signaling [5,14,15,32]. Current evidence does not establish post-COVID bowel dysfunction as a single distinct disease entity. For a patient who develops classic IBS after COVID-19 and meets Rome criteria, COVID-19 is best conceptualized as a potential infectious trigger unless future biomarkers demonstrate a reproducibly distinct pathobiology.

Duration is clinically important. Loose stools lasting days to a few weeks after acute illness can reflect transient recovery or treatment effects. Symptoms persisting for several months deserve evaluation for secondary causes and, when the symptom pattern and chronicity criteria are satisfied, a positive DGBI diagnosis. Prospective Rome-based cohorts show that the number of affected patients generally decreases over time, although a subset has persistent symptoms at six to 12 months [4,15,17].

Clinical Evaluation and Diagnosis

A careful history should establish the temporal relationship to SARS-CoV-2 infection and, critically, whether similar symptoms were present before infection. Stool frequency and form, urgency, nocturnal symptoms, bleeding, mucus, weight loss, fever, dietary triggers, travel, medication and supplement exposure, antibiotics, hospitalization, immune status, family history, hydration, and functional impairment should be documented. Alarm features such as GI bleeding, unexplained anemia or weight loss, fever, severe or progressive pain, nocturnal diarrhea, abnormal examination, or age/family-history concerns should lower the threshold for endoscopy, imaging, or specialist evaluation [6,12,33,34].

In otherwise stable patients with a chronic watery-diarrhea phenotype and no alarm features, testing should be targeted rather than indiscriminate. Guideline-supported components include celiac serology and fecal inflammatory markers such as calprotectin or lactoferrin when inflammatory bowel disease is a concern; Giardia testing and evaluation for bile-acid diarrhea are appropriate in relevant chronic watery-diarrhea presentations, while *C. difficile* and other infectious testing should be guided by antibiotic, healthcare, travel, immune, and epidemiologic risk [33,34]. Additional studies are selected by phenotype as follows: colonoscopy with biopsies when microscopic colitis or IBD is suspected; pancreatic evaluation for steatorrhea or malabsorption; endocrine testing when clinically indicated; and imaging/endoscopy for alarm or unexplained persistent symptoms. Medication-associated diarrhea, bile-acid diarrhea, microscopic colitis, celiac disease, pancreatic insufficiency, persistent infection, IBD, malabsorption, and neoplasia should not be overlooked simply because symptoms followed COVID-19. Figure 2 summarizes a structured clinical approach to evaluating persistent, recurrent, or new-onset diarrhea following COVID-19.

**Figure 2:**
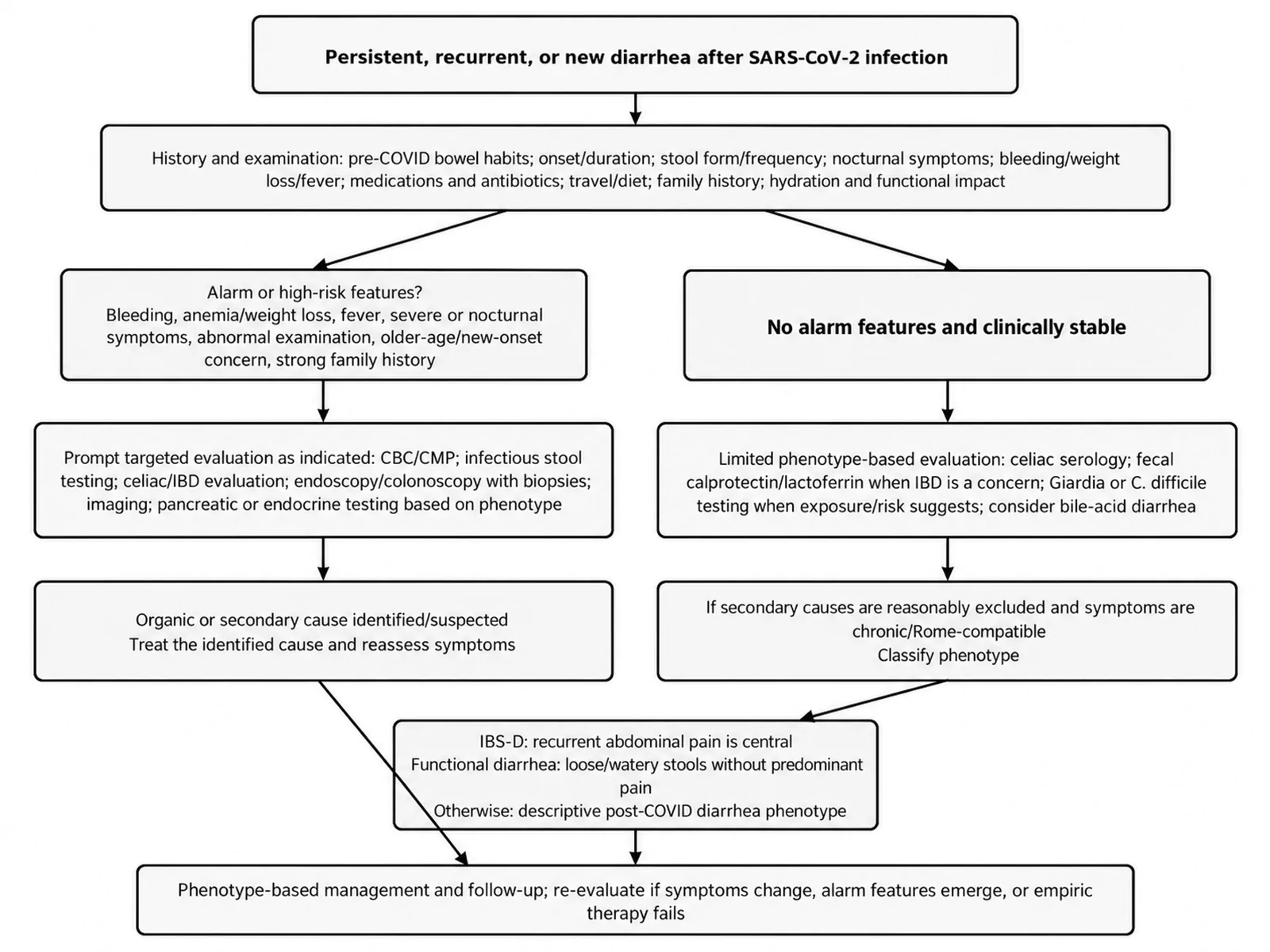
Structured diagnostic approach to persistent or new-onset diarrhea after COVID-19. Testing should be individualized; the algorithm distinguishes routine phenotype-based evaluation from investigations prompted by alarm features or specific suspicion. CMP: comprehensive metabolic panel; IBD: inflammatory bowel disease; IBS-D: diarrhea-predominant irritable bowel syndrome

Therapeutic evidence and management

What Has Actually Been Studied in COVID-19 Populations

Post-COVID-specific intervention evidence is limited. The strongest post-acute randomized evidence is the RECOVERY trial of SIM01, a synbiotic administered at 10 billion colony-forming units per sachet twice daily for six months to 463 patients with post-acute COVID syndrome. Compared with placebo, SIM01 improved several broad symptom domains, including “gastrointestinal upset” (OR: 1.995, 95% CI: 1.304-3.051), with similar overall adverse-event rates (10% versus 11%) [35]. The trial did not specifically enroll patients with chronic diarrhea, functional diarrhea, IBS-D, or another Rome-defined bowel disorder. It therefore supports further study of microbiome modulation in post-acute COVID, but is not direct evidence for a standard treatment of post-COVID functional diarrhea.

Other randomized GI-directed studies were conducted primarily during acute COVID-19. Horvath et al. randomized 30 outpatients with acute COVID-19 to a 10-strain probiotic (at least 5 billion organisms per 5-g portion, twice daily) or placebo for 30 days; 26 completed the study, and clinical recovery and GI symptoms improved over time irrespective of intervention [26,36]. Ivashkin et al. randomized 200 hospitalized patients to standard care with or without a four-strain probiotic (approximately 10^9^ CFU of each strain three times daily for up to 14 days) [27]. Diarrhea present on admission resolved faster with probiotic treatment (median two versus four days; p=0.049), and hospital-acquired diarrhea was lower in the subgroup receiving a single antibiotic, but major COVID-19 outcomes were unchanged [37]. These trials address acute illness, not chronic post-COVID DGBIs.

A randomized double-blind trial of fecal microbiota transplantation (FMT) in 40 patients with mild-to-moderate acute COVID-19 reported improvement in diarrhea, with diarrhea decreasing from 19 patients to zero by day seven in the FMT arm and a between-group difference at day seven (p=0.047) [38]. This finding is clinically interesting but does not establish efficacy for post-acute functional diarrhea. Earlier small uncontrolled FMT reports in convalescent COVID-19 patients are hypothesis-generating rather than confirmatory [24]. Key intervention studies relevant to diarrhea or gastrointestinal symptoms in COVID-19 are summarized in Table 4.

**Table 4:** Intervention studies relevant to diarrhea or GI symptoms in COVID-19. PACS: post-acute COVID-19 syndrome; AEs: adverse events; AAD: antibiotic-associated diarrhea; FMT: fecal microbiota transplantation; DGBI: disorders of gut-brain interaction

Studies	Population	Intervention/duration	Comparator	GI-specific result/safety	Relevance to post-COVID diarrhea
Lau et al., SIM01 [25]	Post-acute COVID syndrome; n=463	SIM01 synbiotic, 10 billion CFU/sachet twice daily for 6 months	Placebo	Broad PACS symptom alleviation; GI upset OR: 1.995 (1.304-3.051); AEs 10% versus 11%	Post-acute, but not a defined chronic-diarrhea/Rome-positive trial
Horvath et al. [26]	Acute outpatient COVID; 30 randomized, 26 completed	10-strain OMNi-BiOTiC 10/Ecologic AAD; ≥5 billion organisms per 5-g portion; twice daily for 30 days	Placebo	No clear intervention-specific clinical or GI symptom benefit; microbiome changed; no attributable serious safety signal	Acute disease; very small trial; not post-COVID diarrhea
Ivashkin et al. [27]	Hospitalized acute COVID pneumonia; n=200	*L. rhamnosus* PDV 1705, *B. bifidum* PDV 0903, *B. longum* subsp. infantis PDV 1911, *B. longum* subsp. longum PDV 2301; ~10^9^ CFU each, 3x/day, up to 14 days	Standard care	Admission diarrhea resolved in 2 versus 4 days (p=0.049); subgroup reduction in hospital-acquired diarrhea; no major outcome benefit	Acute inpatient evidence; antibiotic exposure and open-label design limit extrapolation
Jiang et al., FMT [35]	Mild-moderate acute COVID; n=40	Encapsulated donor FMT; short course	Placebo capsules	Diarrhea improved; 19 to 0 by day 7 in FMT arm, between-group p=0.047	Acute COVID; small trial; does not establish post-acute DGBI treatment

Conventional Symptom-Directed Management: Extrapolated, Not Post-COVID-Proven

When organic disease has been reasonably excluded, and the phenotype is functional diarrhea or IBS-D, conventional management can follow established GI guidelines, with the important caveat that these strategies have generally not been validated in dedicated post-COVID trials [33,34]. Hydration and electrolyte correction are first-line when clinically relevant. Medication review is essential because antibiotics, metformin, magnesium-containing products, laxatives, and other agents can cause or perpetuate diarrhea. Dietary interventions should be individualized; soluble fiber and a time-limited low-FODMAP trial can be considered for an IBS phenotype, ideally with nutritional guidance when restriction is substantial [33]. Loperamide may be used for troublesome stool frequency/urgency in appropriate patients; bile-acid sequestrants are reserved for suspected or demonstrated bile-acid diarrhea; antispasmodics or neuromodulators may be selected for pain-predominant DGBI phenotypes. These are evidence-informed GI treatments, not disease-modifying therapies for long COVID.

Probiotics, FMT, and Microbiome Restoration

Probiotic effects are strain- and formulation-specific. The SIM01 synbiotic, the 10-strain OMNi-BiOTiC formulation, and the four-strain PDV formulation should not be treated as therapeutically interchangeable [35-37]. No single probiotic strain, dose, or treatment duration can currently be recommended as a standard therapy for post-COVID functional diarrhea. Likewise, commercial microbiome tests do not yet provide validated individualized treatment selection for this indication [25].

FMT is even more investigational in this setting. Although the small acute-COVID randomized trial showed a diarrhea signal, durability, optimal donor selection, long-term safety, and efficacy in chronic post-COVID diarrhea remain unknown [38]. FMT can transmit infectious or multidrug-resistant organisms, making rigorous donor screening and regulated processing essential. Current AGA guidance supports fecal microbiota-based therapies for selected recurrent or severe/fulminant *Clostridioides difficile* scenarios and advises against conventional FMT for IBS outside clinical trials [39]. Post-COVID diarrhea therefore should not be treated with FMT as routine care; use should be limited to appropriately regulated research or another accepted indication [39,40].

Emerging Targets and Biomarkers

Candidate therapeutic pathways include epithelial barrier repair, microbial SCFA restoration, bile-acid signaling, persistent-antigen responses, immune modulation, and neuroimmune signaling [18,19,25,28,31]. Human evidence is currently strongest for associative biomarkers rather than validated treatment targets as follows: compositional microbiome patterns, SCFA biosynthetic capacity, inflammatory markers, and selected metabolomic signatures correlate with acute severity or broad PACS phenotypes [18,28,30,31]. None currently distinguishes a clinically validated “viral-persistence diarrhea,” “dysbiosis diarrhea,” or other mechanistic subtype for routine care. Biomarker-driven treatment remains a research goal.

Implications for public health and future research

Screening, Surveillance, and Prevention

Because SARS-CoV-2 infection has been widespread, even modest excess GI risk can generate meaningful clinical burden [1,2]. It is reasonable for follow-up encounters to ask about new bowel changes, bleeding, weight loss, hydration, and functional impairment, particularly after an acute illness with GI symptoms. This is a pragmatic clinical screening recommendation rather than evidence that population-wide GI screening prevents post-COVID DGBIs.

Evidence specifically showing that vaccination, dietary modification, probiotics, exercise, sleep optimization, or psychosocial interventions prevent post-COVID functional bowel disorders is insufficient. Vaccination is evidence-based for prevention of severe acute COVID-19 and may reduce some post-acute outcomes, but prevention of a specific bowel DGBI has not been established. Antibiotic stewardship, adequate nutrition, physical activity as tolerated, sleep, and psychosocial support should therefore be described as general health and risk-reduction measures rather than proven prevention strategies for post-COVID diarrhea.

Priority Research Questions

Future research should (1) use standardized definitions that distinguish post-COVID symptoms from Rome-defined DGBIs; (2) document pre-infection GI status prospectively; (3) include both uninfected controls and, when feasible, non-COVID infectious controls; (4) record vaccination, variant/wave, acute severity, hospitalization, antibiotics, medications, diet, and healthcare access; (5) collect validated symptom, quality-of-life, and work-function outcomes longitudinally; (6) integrate stool taxonomy with functional measures such as SCFAs, bile acids, tryptophan pathways, and other metabolites; (7) pair serial stool/blood sampling with mucosal studies in selected cohorts to test directionality between antigen persistence, barrier injury, inflammation, and symptoms; and (8) conduct adequately powered randomized trials in well-phenotyped populations such as persistent diarrhea, functional diarrhea, or IBS-D rather than broad PACS alone. These design priorities are needed to move from association to clinically actionable mechanisms [1-4,18,19,21,22,25,28,31,35].

Limitations of the evidence and of this narrative review

The underlying literature is heterogeneous in definitions of long COVID, symptom ascertainment, control groups, follow-up duration, vaccination and variant era, acute disease severity, and pre-infection GI documentation. Administrative datasets provide scale but may misclassify functional diagnoses; survey studies are vulnerable to recall and response bias; mechanistic microbiome studies are frequently small and confounded; and many intervention trials address acute COVID-19 or broad PACS rather than chronic diarrhea. Publication bias is plausible because positive post-COVID associations may be more likely to be reported.

This review also has methodological limitations. It is a narrative synthesis with targeted literature updating rather than a prospectively registered systematic review. Study selection was not performed by independent duplicate reviewers, no formal risk-of-bias instrument was applied to each study, and no meta-analysis or certainty-of-evidence grading was conducted. Manual study selection may introduce selection bias, and the review may not capture every relevant publication despite the September 2026 update. Quantitative estimates are therefore presented to characterize the evidence, not to generate a new pooled effect size.

## Conclusions

Post-COVID GI symptoms are real and clinically relevant, but persistent diarrhea should not be equated automatically with a functional bowel disorder. Established evidence shows an excess post-acute burden of GI symptoms and diagnoses after SARS-CoV-2 infection and prospective development of Rome-defined DGBIs in a subset of patients. It is also established that the intestine can be involved during acute infection and that post-acute microbial and biologic abnormalities can persist in selected cohorts.

What remains unproven is equally important: persistent viral RNA or antigen is not the same as replication-competent virus; dysbiosis has not been established as the sole cause of chronic diarrhea; gut-lung signaling is a proposed pathway; and microbiome-directed interventions have not been validated as disease-modifying therapy for post-COVID functional diarrhea. The most appropriate current strategy is a phenotype-based clinical evaluation that excludes important organic, infectious, medication-related, and metabolic causes, followed by established DGBI management when diagnostic criteria are met. Dedicated, well-controlled post-COVID diarrhea trials are needed before specific microbiome, antiviral, immune, or barrier-directed therapy can be recommended.
